# Real-world monitoring strategies and predictors guiding the transition from active surveillance to treatment in ISUP 1 prostate cancer

**DOI:** 10.1007/s00432-026-06441-9

**Published:** 2026-03-04

**Authors:** Giulia Giannini, Amer Mousa, Eberhard Steiner, Nastasiia Artamonova, Mona Kafka, Isabel Heidegger

**Affiliations:** Department of Urology, Medical University of Innsbruck, Anichstreet 35, 6020 Innsbruck, Austria

**Keywords:** Active surveillance, ISUP 1, Monitoring, Prostate cancer, Real world setting, Predictors for active therapy

## Abstract

**Purpose:**

Active surveillance (AS) is the standard approach for managing ISUP 1 prostate cancer (PCa), ensuring oncological safety while minimizing overtreatment. However, AS protocols vary significantly. This study assessed a structured AS protocol incorporating PSA kinetics, multiparametric MRI (mpMRI), and targeted biopsies to identify predictors of treatment transition.

**Methods:**

We retrospectively reviewed 89 patients with ISUP 1 PCa managed with AS between 2010 and 2024. Inclusion criteria were PSA < 10 ng/mL and ≤ 2 positive biopsy cores with ≤ 50% tumor involvement per core. Patients underwent regular PSA testing, mpMRI, and risk-adapted biopsies. Outcomes included treatment conversion, progression predictors, and the role of mpMRI.

**Results:**

Median follow-up was 52.8 months. PSA was monitored at a median interval of 4.8 months, and mpMRI every 15.7 months (median). At diagnosis, 68.5% underwent mpMRI, with PIRADS 4 as the most frequent. During AS, 66.3% underwent at least one re-biopsy, most commonly triggered by PSA progression (73%). Treatment was initiated in 37.1% after a median of 37.9 months due to PSA progression (54.5%), mpMRI changes (21.2%), combined PSA/mpMRI findings (6.1%), histological upgrading (9.1%), or patient preference (3%). Treatments included radical prostatectomy, EBRT or LDR brachytherapy. Among these patients, no PSA persistence was observed; biochemical recurrence occurred in 6.1%, metastases in 4.0%, and overall mortality in 4.5%, with no PCa-specific deaths.

**Conclusion:**

A structured, risk-adapted AS protocol using PSA kinetics and mpMRI enabled personalized management and timely detection of progression. These findings support the standardization of AS protocols, which should be validated in larger cohorts.

## Introduction

Prostate cancer (PCa) is the most frequently diagnosed solid malignancy in men, with highly heterogeneous clinical behavior (Cornford et al. 2024). Active surveillance (AS) is the standard approach for managing low-risk PCa, balancing the risks of overtreatment with the need for timely intervention (Kinsella et al. 2018). Long-term data from large AS cohorts, such as ProtecT and PRIAS, have demonstrated that AS offers prostate cancer-specific survival rates comparable to radical treatments, reinforcing its role as a safe alternative for well-selected patients (Bul et al. 2013; Willemse et al. 2022).

Despite widespread adoption, AS protocols vary significantly in monitoring strategies and progression criteria. Some studies, such as PRIAS follow fixed biopsy schedules, while others favor a risk-adapted approach incorporating PSA kinetics, multiparametric MRI (mpMRI), and targeted biopsies (Bul et al. 2013; Lee et al. 2021). mpMRI has improved patient selection and progression detection, but standardized MRI-based protocols are lacking (Lee et al. 2021).

A further challenge is the absence of standardized criteria for progression. While PSA kinetics are commonly used to guide AS decisions, their reliability has been questioned due to its natural variability, which may not accurately reflect oncological progression (Taneja 2015) additionally, some patients discontinue AS despite no objective disease worsening, often due to anxiety or uncertainty (Sypre et al. 2022; Taneja 2015). In this context, incorporating biomarkers such as genomic classifiers (e.g., Prolaris, Decipher) may further enhance risk stratification and optimize treatment decisions.

This study aimed to evaluate outcomes in patients managed with a flexible AS protocol incorporating PSA monitoring, mpMRI, and targeted biopsies, specifically addressing: 1) predictors of transition to active treatment; 2) the role of mpMRI in AS management; and 3) oncological outcomes, including upgrading, biochemical recurrence (BCR), and metastases.

## Patients and methods

We retrospectively reviewed 89 patients diagnosed with International Society of Pathology 1 (ISUP 1) PCa who underwent AS between 2010 and 2024, following approval from the local ethical committee (vote number:1296/2923). Inclusion criteria were: (1) histologically confirmed ISUP 1 PCa, (2) PSA < 10 ng/mL, (3) ≤ 2 positive biopsy cores with ≤ 50% tumor involvement per core, and (4) scheduled monitoring with PSA, mpMRI, and re-biopsies. These inclusion criteria remained consistent throughout the entire study period (2010–2024). Patient selection and AS management were aligned with contemporary EAU guideline recommendations and established cohorts such as PRIAS, while allowing flexibility according to individual patient risk in a real-world setting (Bul et al. 2013). According to institutional practice, PSA monitoring was generally planned every 3–6 months, while mpMRI was intended to be performed approximately annually. PSA monitoring represented a central component of the surveillance strategy and was used not only for longitudinal assessment but also to guide further diagnostic steps. PSA progression was predefined using a hierarchical approach reflecting real-world clinical practice: PSA > 10 ng/mL, PSA doubling time (PSA-DT) < 12 months, or a suspicious PSA trend when PSA-DT could not be reliably calculated defined as three consecutively rising PSA values with PSA < 10 ng/mL (Bul et al. 2013; Hamdy et al. 2023; Tosoian et al. 2016). The PSA > 10 ng/mL threshold reflects commonly adopted exit criteria in contemporary active surveillance cohorts and guideline-based practice, aiming to balance oncological safety with avoidance of overtreatment. Routine use of mpMRI became established from 2018 onwards, following its wider availability in clinical practice. mpMRI examinations were interpreted according to the PIRADS version in use at the time of imaging and were reviewed by experienced uro-radiologists in a high-volume center. Specifically, PIRADS version 1 was used in the early years of the study, followed by PIRADS v2 and v2.1 after their respective introduction into clinical practice, without retrospective re-scoring. mpMRI of the prostate was performed using 1.5 T or 3 T scanners, according to standardized institutional protocols consistent with contemporaneous PIRADS recommendations. The mpMRI protocol included high-resolution T2-weighted imaging, diffusion-weighted imaging, and dynamic contrast-enhanced sequences when available.

In this real-world cohort, follow-up intervals were adapted based on individual clinical characteristics, PSA kinetics, imaging findings, and patient adherence, rather than being applied at strictly fixed time points. Clinical data were extracted from electronic records and included demographics, PSA values, mpMRI findings, and biopsy results. Re-biopsies were performed according to predefined clinical criteria and were triggered by PSA progression and/or mpMRI progression. All diagnostic biopsies were performed using a transrectal ultrasound-guided approach, with a median of 15 biopsy cores per procedure. A transperineal approach was not routinely adopted during the study period. Transition to active treatment was based on histological upgrading, PSA progression, mpMRI progression, or patient choice. Over the 14-year study period, the AS protocol evolved, particularly with increasing standardization of PSA monitoring and a more frequent use of mpMRI following its wider adoption in clinical practice. Re-biopsy decisions were not scheduled at fixed intervals but were based on predefined clinical criteria.

## Statistical analysis

Continuous variables were described using median and interquartile range. Categorical variables were compared using the chi-square test, while continuous variables were assessed with the Student’s t-test or Mann–Whitney U test. These statistical tests were used to perform descriptive comparisons between patients who remained on AS and those who transitioned to active treatment. Statistical significance was set at *p* < 0.05. Analyses were performed using Python (pandas, scipy, statsmodels). Given the limited sample size and number of events, analyses of predictors of transition to active treatment were exploratory and descriptive in nature, and the study was not powered to perform formal time-to-event analyses such as Kaplan–Meier or Cox proportional hazards models.

## Results

A total of 89 patients were included in the AS regime, with a median follow-up of 52.8 months (range: 7.23–169.3 months). Median age at diagnosis was 67.8 years (range: 52–86), BMI was 27.2 kg/m^2^ (range: 14.7–37.6) and prostate volume was 52.5 mL (range: 39.6–67.5 mL). Prior to diagnosis, 14.6% had a tumor negative biopsy (Table 1). Reliable data on the number of patients initially assessed but excluded were not available due to the retrospective study design.

**Table 1:** Baseline patient characteristics and mpMRI findings

Variable	Value
Number of patients	89
Follow-up duration, months, median (range)	52.8 (7.23–169.3)
*Diagnosis modality*	
Biopsy-detected PCa	94.4% (n = 84)
Incidental PCa	5.6% (n = 5)
Age at diagnosis, years, median (range)	67.8 (52–86)
Prostate volume, mL, median (range)	52.5 (39.6–67.5)
PSA density at diagnosis, median (range)	0.108 (0.016–0.277)
PSA density ≥ 0.15 ng/mL^2^f	21 (23.1%)
Body mass index, kg/m^2^, median (range)	27.2 (14.7–37.6)
*BMI category*	
Normal weight (BMI < 25)	41.6% (n = 37)
Overweight (BMI 25–29.9)	35.9% (n = 32)
Obese (BMI ≥ 30)	13.3% (n = 12)
Underweight / unknown	9.2% (n = 8)
Smoking status (yes / no / unknown)	7.9% / 74.9% / 17.2%
Previous negative biopsy	14.6% (n = 13)
mpMRI performed before biopsy (yes / no / unknown)	68.5% / 30.3% / 1.1%
*PIRADS distribution (n* = *61)*	
PIRADS 2	1.6% (n = 1)
PIRADS 3	9.9% (n = 6)
PIRADS 4	80.3% (n = 49)
PIRADS 5	8.2% (n = 5)
*Lesion distribution (n* = *58 patients)*	
Single lesion	89.7% (n = 52)
Multifocal disease	10.3% (n = 6)
*Lesion location by zone (n* = *66 lesions)*	
Apex	~ 32%
Peripheral zone	~ 28%
Transition zone	~ 18%
Other / anterior / central	~ 22%

*PCa* Prostate Cancer, *BMI* Body Mass Index, *PSA* Prostate-specific antigen, *mpMRI* multiparametric magnetic resonance imaging, *PI-RADS* Prostate Imaging Reporting and Data System

mpMRI was performed before biopsy in 61 patients (68.5%). Among these, 67.2% (n = 41) were diagnosed in 2018 or later, when mpMRI became recommended. Notably, 32.8% (n = 20) underwent mpMRI despite being diagnosed before 2018. Among patients without mpMRI (30.3%, n = 27), only 14.8% (n = 4) were diagnosed after 2018, suggesting that most of these cases predate routine mpMRI use. mpMRI status was unknown in one patient (1.1%) (Table 1).

Among the 58 patients with mpMRI-detectable lesions, 66 lesions were identified. PIRADS scores were: PIRADS 2 in 1.6%, PIRADS 3 in 9.9%, PIRADS 4 in 80.3%, and PIRADS 5 in 8.2%. Most lesions were located in the apex, peripheral, and transition zones; the full distribution is reported in Table 1. Notably, 89.7% had a single lesion, while 10.3% presented with multifocal disease.

PSA was monitored at a median interval of 4.8 months (range: 3–12 months). 45% of patients had PSA measurements every 3 to 4 months, while 55% had intervals of six months or longer. mpMRI scans were conducted at a median interval of 15.7 months (range: 4–61 months), and repeat biopsies occurred on median every 22.9 months (range: 5.9–96.9 months). The variability in PSA and mpMRI monitoring intervals reflects a risk-adapted follow-up strategy, in which monitoring intensity was tailored according to individual disease characteristics and PSA kinetics rather than fixed time points. In addition, the wide range of monitoring intervals reflects the evolution of the AS protocol over the 14-year study period and variable patient adherence inherent to a real-world clinical setting.

PSA progression occurred in 52.8% of patients (n = 47), based on predefined hierarchical criteria: (1) PSA > 10 ng/mL, (2) PSA-DT < 12 months, or (3) a suspicious PSA trend defined as three consecutively rising values with PSA < 10 ng/mL and non-calculable PSA-DT. The definition of PSA progression was based on predefined internal criteria reflecting real-world clinical practice and was consistent with PSA kinetics and PSA rise thresholds commonly used for clinical reassessment in major AS and active monitoring cohorts, although no universally standardized definition exists (Bul et al. 2013; Hamdy et al. 2023; Tosoian et al. 2016). Specifically, 24.7% had PSA > 10 ng/mL, 4.5% both PSA > 10 and PSA-DT < 12 months, 7.9% had PSA-DT < 12 months without elevated PSA, and 15.7% met only the trend criterion. Additionally, 22.5% had a PSA-DT between 12 and 24 months, 10.1% > 24 months, and in 25.8% PSA-DT could not be calculated due to insufficient or irregular measurements.

The median time to PSA progression was 27.3 months (range: 0.7–130.6 months). Re-biopsies were performed in 66.3% of patients (n = 59). Indications included PSA progression alone in 23 patients (39.0%), combined PSA progression and mpMRI changes in 24 (40.6%), and mpMRI progression without PSA changes in 9 (15.2%). One patient (1.7%) was re-biopsied due to missing baseline mpMRI, and in two cases (3.3%) no clear clinical or radiological indication was identified. Biopsies were not scheduled at fixed intervals but were performed based on clinical judgment, with a median interval of 22.9 months (range: 5.9–96.9 months) **(**Table 2**)**.

**Table 2:** Clinical parameter during active surveillance

Variable	Value
PSA monitoring interval, months, median (range)	4.8 (3–12)
mpMRI monitoring interval, months, median (range)	15.7 (4–61)
PSA progression (overall)	52.8% (n = 47)
Main trigger for PSA progression*	
PSA > 10 ng/mL	24.7% (n = 22)
PSA-DT < 12 months	7.9% (n = 7)
Suspicious PSA trend	15.7% (n = 14)
Combined PSA > 10 ng/mL and PSA-DT < 12 months	4.5% (n = 4)
No PSA progression	47.2% (n = 42)
*PSA doubling time distribution*	
PSA-DT 12–24 months	13.5% (n = 12)
PSA-DT > 24 months	10.1% (n = 9)
PSA-DT not calculable	23.6% (n = 21)
Re-biopsy performed during AS	66.3% (n = 59)
*Indication for re-biopsy*	
PSA progression alone	39.0% (n = 23)
Combined PSA progression and mpMRI changes	40.6% (n = 24)
mpMRI progression without PSA changes	15.2% (n = 9)
Other / unclear indication**	5.1% (n = 3)

*PSA* Prostate-specific antigen, *mpMRI* multiparametric magnetic resonance imaging, *PSA-DT* PSA-Doubling Time

^*^Suspicious PSA trend: defined as three consecutively rising PSA values, all < 10 ng/mL, in the absence of a calculable PSA doubling time (PSA-DT), often due to ≥ 6-month intervals between measurements. This definition aligns with previous active surveillance literature (e.g., Klotz et al., JCO 2015; Tosoian et al., JCO 2016)

^**^Includes re-biopsy due to missing baseline mpMRI or without a clearly identifiable clinical or radiological indication

Overall, 33 patients (37.1%) transitioned to active therapy after a median duration of 37.9 months (range 10–116.9). Although histological upgrading is recommended before definitive treatment according to EAU Guidelines, in our cohort the decision to initiate active treatment was based on a combination of clinical factors, including PSA kinetics, mpMRI progression, urinary symptoms, and patient preference, within a shared decision-making framework. PSA progression alone was the indication in 18 patients (54.5%), mpMRI changes without PSA abnormalities in 7 (21.2%), and combined PSA/mpMRI progression in 2 (6.1%). Histological upgrading prompted treatment in 3 cases (9.1%). The remaining 3 patients (9.1%) lacked a clear clinical or radiological indication; one case reflected patient preference. Urinary obstructive symptoms were more frequent in treated patients (28.6%) compared to those remaining in AS (14.3%, p = 0.02) **(**Table 3**)**.

**Table 3:** Reasons for transition to active treatment, outcomes and follow-up

Variable	Value
Patients transitioning to active treatment	33 (37.1%)
Time on Active Surveillance, months, median (range)	37.9 (10–116.9)
*Primary reason for transition*	
PSA increase alone	54.5% (n = 18)
mpMRI changes alone	21.2% (n = 7)
Combined PSA increase and mpMRI changes	6.1% (n = 2)
Histological upgrading on re-biopsy	9.1% (n = 3)
Patient preference	3.0% (n = 1)
Obstructive urinary symptoms (treated vs AS)	28.6% vs 14.3% (p = 0.02)
*Treatment modality*	
Radical prostatectomy	69.7% (n = 23)
LDR brachytherapy	9.1% (n = 3)
External beam radiotherapy (EBRT)	21.2% (n = 7)
*Pathological stage after radical prostatectomy (n* = *23)*	
pT2 (overall)	91.3% (n = 21)
pT2a	38.0% (n = 8)
pT2b	9.5% (n = 2)
pT2c	52.5% (n = 11)
pT3b	4.3% (n = 1)
Unknown	4.3% (n = 1)
*Post-treatment outcomes*	
PSA persistence	0% (n = 0)
Biochemical recurrence (BCR)	6.1% (n = 2)
Post-treatment follow-up, months, median (range)	22.1 (0.1–102.1)
Metastatic disease	4.0% (n = 1)
Overall mortality	5.6% (n = 5)
Prostate cancer–specific mortality	0%

*PSA* Prostate-specific antigen, *mpMRI* multiparametric magnetic resonance imaging, *AS* active surveillance, *TNM* TNM Classification of Malignant Tumors *PSA* Prostate-specific antigen, *BCR* Biochemical Recurrence

Among treated patients, 23 patients (69.7%) underwent radical prostatectomy (RP), 3 patients (9.1%) received an LDR-brachytherapy, and 7 patients (21.2%) underwent external beam radiation therapy (EBRT).

Advanced pathological stage occurred in a single patient. At diagnosis, PSA was 8.8 ng/mL and baseline mpMRI showed a suspicious lesion (PIRADS 4). During AS, follow-up consisted of serial PSA testing and repeated mpMRI examinations; no repeat biopsy was performed. PSA values during follow-up showed marked fluctuations, with repeated increases above 10 ng/mL (11.24 ng/mL in 2018 and 12.8 ng/mL in 2021). PSA monitoring intervals were irregular, with prolonged gaps between measurements, reportedly related to patient decision, limiting reliable PSA doubling time calculation.

The first mpMRI was performed relatively late, approximately 23 months after diagnosis. Overall, the patient underwent three mpMRI examinations during AS, subsequently performed at intervals of approximately 8–9 months. The latter mpMRI examinations showed radiological progression of the lesion. Definitive treatment was initiated approximately 3–6 months after the last PSA increase above 10 ng/mL and in the context of radiological progression.

Pathology showed pT2 in 91.3% (38% pT2a, 9.5% pT2b, 52.5% pT2c), and pT3b in 4.3%; one case was unknown. No patients experienced PSA persistence post-treatment, and two patients developed a BCR. After a median post-treatment follow-up of 22.1 months (range: 0.1–102.1), 4.0% developed metastases and overall mortality was 5.6%, with no PCa-specific deaths **(**Table 3**)**.

## Discussion

AS is increasingly recognized as a safe and effective treatment strategy for managing ISUP 1 PCa, particularly in low-risk patients (Willemse et al. 2022). Long-term data from PRIAS, ProtecT, and Tosoian et al. confirm its oncological safety, with survival outcomes comparable to radical treatments (Alberti et al. 2024; Bul et al. 2013; Tosoian et al. 2016). The novelty of this study lies in the detailed description of real-world decision-making during AS, highlighting how PSA kinetics and mpMRI findings are integrated in daily clinical practice outside rigid trial-based protocols. In our study, this approach translated into a selective follow-up strategy in which re-biopsies were performed based on PSA kinetics and mpMRI findings, with only 66.3% of patients undergoing re-biopsy during follow-up. Long-term data from large AS cohorts, including the PRIAS consortium, demonstrate that while histological upgrading is relatively common in men with Grade Group 1 prostate cancer, the risk of metastatic progression remains very low (de Vos et al. 2025). The ProtecT trial, in particular, found no significant difference in prostate cancer-specific mortality among AS, RP, and EBRT after 15 years of follow-up (Alberti et al. 2024).

PRIAS mandates biopsies at fixed intervals (Bul et al. 2013) whereas other programs promote risk-adapted monitoring, integrating PSA kinetics and mpMRI findings (Padhani et al. 2021). Our study contributes to this evolving landscape by supporting the concept that a flexible AS protocol with risk-based re-biopsy may reduce unnecessary interventions while preserving safety.

Although monitoring was tailored to each patient, we applied a structured framework to minimize biopsies without compromising oncological control. PSA was measured every 4.8 months, mpMRI every 15.7 months, and biopsies were performed selectively (median 2–3 per patient). In contrast, PRIAS mandates biopsies at 1, 4, 7, and 10 years, regardless of clinical findings (Bul et al. 2013). We acknowledge that diagnostic biopsies in our cohort were performed using a transrectal approach, with systematic sampling and MRI/ultrasound fusion guidance when mpMRI-visible lesions were present. Nevertheless, recent evidence suggests that transperineal saturation biopsy combined with mpMRI may further reduce the risk of disease reclassification during AS (Pepe et al. 2022). Their definition of PSA fluctuations, described as variations without clear trends, differs from our more stringent criterion which required three consecutively rising PSA values despite PSA < 10 ng/mL and non-calculable PSA-DT (Ciccone et al. 2023). In our study, the use of stricter PSA progression criteria was associated with fewer re-biopsies compared to cohorts applying less clearly defined PSA fluctuation thresholds. However, the present study was not designed to formally assess a reduction in unnecessary interventions or oncological safety, and these aspects require confirmation in larger cohorts with standardized follow-up.

Taneja et al. noted the limitations of PSA alone in detecting progression and the lack of standardized criteria across protocols (Taneja 2015). Among urinary biomarkers, PCA3 has shown potential in refining prostate cancer diagnosis, particularly in repeat biopsy settings, although its role in contemporary AS remains complementary to mpMRI rather than decisive (Pepe & Aragona 2011). This highlights the importance of incorporating multiple monitoring parameters to minimize unnecessary biopsies and enhance the accuracy of disease progression assessments (Taneja 2015). In this context, molecular classifiers (e.g., Prolaris, Decipher) and urinary biomarkers (e.g., SelectMDx, PCA3) are gaining interest as tools to enhance risk stratification (Deluce et al. 2022; Shore et al. 2014; Wei 2015). Walker et al. highlighted the marked variability in AS protocols across institutions, particularly regarding biopsy schedules (Walker et al. 2022). In men on AS without radiological signs of disease progression on mpMRI, PSA density has been reported to add information for risk stratification, identifying patients at higher risk of reclassification despite stable imaging findings (Roscigno et al. 2020). In our real-world cohort, PSA density was not used as a formal criterion to guide AS management or treatment decisions but is reported here as a descriptive baseline parameter. Future prospective studies are warranted to clarify the role of PSA density in combination with mpMRI findings and to define standardized thresholds for clinical decision-making in AS protocols. Some centers, such as Johns Hopkins (JHU) and PASS, perform annual biopsies, while PRIAS follows a rigid schedule, and the EAU guidelines recommend biopsies only in cases of clinical progression (Walker et al. 2022).

Chau Hung Lee et al. highlighted the central role of mpMRI in AS, showing that up to 40% of patients initially eligible based on clinical criteria were reclassified due to imaging findings (ISUP ≥ 2) (Lee et al. 2021). This underscores the value of mpMRI in avoiding underestimation of disease. They also described the PRECISE scoring system, which classifies radiological stability or progression from 1 to 5, prompting biopsy in cases scored 4–5. (Lee et al. 2021). In contrast, our study did not formally apply the PRECISE system, but we used a combined approach integrating PSA-DT, mpMRI findings, and targeted biopsies to monitor disease status. Given the long study period, mpMRI findings in our cohort were interpreted using different PIRADS versions over time, which reflects routine clinical practice rather than a limitation of the surveillance approach. In our study, disease monitoring relied on the combined assessment of PSA-DT, mpMRI findings, and targeted biopsies, reflecting a pragmatic real-world approach.

In our study, re-biopsies were performed based on predefined progression indicators, including continuously rising PSA, PSA-DT < 12 months, PSA > 10 ng/mL, new mpMRI lesions, or significant lesion growth. Emerging evidence suggests that PSMA PET/CT may further refine risk stratification in men on AS, potentially identifying clinically significant disease not detected by mpMRI alone (Pepe et al. 2023).

Padhani et al. (2021) analyzed the role of mpMRI in guiding biopsy decisions during AS and observed that if mpMRI findings alone were used, approximately two-thirds of patients could avoid biopsy based on negative or stable imaging. However, this strategy would miss histological upgrading in nearly one-third of progressing cases.

The transition to active therapy is a critical endpoint. In our study, 37.1% of patients transitioned to active therapy after a median of 37.9 months This rate is comparable to Klotz et al. (36% at 10 years, 45% at 15 years) and Tosoian et al. (50% at 10 years, 57% at 15 years) (Klotz et al. 2015; Tosoian et al. 2016), but higher than PRIAS (21.1%) and lower than Ciccone et al. (47% at 48 months) (Bul et al. 2013; Ciccone et al. 2023).

Davidson Sypre et al. found no significant increase in anxiety or depression among AS patients compared to those treated with RP or RT (Sypre et al. 2022). However, in our cohort, 3 patients (9.1%) transitioned to active therapy without documented clinical or radiological progression, and in one case, treatment was explicitly driven by patient preference. This suggests that individual perception of risk may influence decision-making. In our study, urinary obstructive symptoms were also more common among treated patients, aligning with previous findings linking LUTS to AS discontinuation (Alberti et al. 2024; Walker et al. 2022).

At final pathology, 91.3% had pT2 and 4.3% had pT3 disease, with no R1 or pN + cases. These outcomes are consistent with PRIAS, but contrast with ProtecT, where 50.5% had Gleason Grade ≥ 2 and 28.5% had pT3/pT4 tumors (Alberti et al. 2024; Bul et al. 2013). BCR occurred in 6.1% (n = 2), metastases developed in 4.0%, and overall mortality was 5.6%, with no prostate cancer-specific deaths. (Alberti et al. 2024; Bul et al. 2013).

This study has some limitations. Additional limitations include the retrospective single-center design, the relatively small sample size, and variability in PSA and mpMRI monitoring intervals inherent to a real-world setting. The absence of protocol-mandated repeat biopsies reflects the risk-adapted surveillance strategy applied in this cohort; therefore, our findings should be considered hypothesis-generating rather than definitive. The median follow-up of 52.8 months offers robust mid-term data, though shorter than in PRIAS (10 years) and ProtecT (15 years) (Alberti et al. 2024; Bul et al. 2013). However, our follow-up is comparable to that of Ciccone et al. (48 months) (Ciccone et al. 2023). Second, we did not directly assess psychological aspects, but the transition to therapy in a few patients without oncological progression suggests that anxiety or uncertainty may have influenced decisions. Third, molecular biomarkers such as Prolaris or Decipher were not included in risk stratification. While PSA, mpMRI, and biopsies remain standard, genomic classifiers may improve patient selection and reduce overtreatment. Importantly, we are currently conducting a study evaluating the impact of Prolaris on AS decision-making, which may help refine future AS protocols. Fourth, while we employed mpMRI and targeted biopsies to monitor disease progression, we did not formally apply the PRECISE scoring system, which standardizes mpMRI assessment in AS. Instead, progression assessment relied on PSA-DT, imaging, and histological findings. Future research may clarify whether PRECISE adds value to monitoring. Finally, being a single-center study may limit generalizability, though our results align with multicenter AS data.

## Conclusion

AS is a safe and effective strategy for ISUP 1 PCa, with outcomes comparable to long-term AS cohorts. In our study, 37.1% of patients transitioned to active treatment, primarily due to PSA progression (54.5%) or mpMRI changes (21.2%). Only a minority was treated without confirmed progression. These findings underscore the importance of combining mpMRI with PSA kinetics and targeted biopsies in AS protocols. The lack of standardized progression criteria remains a challenge. A risk-adapted approach based on PSA dynamics, imaging, and selective re-biopsy may support clinical decision-making. Future studies should evaluate long-term outcomes and the role of biomarkers to refine risk stratification.

## Data Availability

The data that supports the findings of this study are available from the corresponding author upon reasonable request.
